# Influence of Carbon-Based Fillers on the Electromagnetic Shielding Properties of a Silicone-Potting Compound

**DOI:** 10.3390/ma17020280

**Published:** 2024-01-05

**Authors:** Rafael Seidel, Konrad Katzer, Jakob Bieck, Maurice Langer, Julian Hesselbach, Michael Heilig

**Affiliations:** 1 Fraunhofer Institute of Material and Beam Technology IWS, 01277 Dresden, Germany; 2 SKZ—German Plastics Center, 97076 Wuerzburg, Germany

**Keywords:** electromagnetic interference, shielding, carbon-based fillers, rheology, stripline, conductivity, carbon nanotubes

## Abstract

The effect of carbon-based additives on adhesives and potting compounds with regard to electrical conductivity and electromagnetic interference (EMI) shielding properties is of great interest. The increasing power of wireless systems and the ever-higher frequency bands place new demands on shielding technology. This publication gives an overview of the effect of carbon-based fillers on electrical conductivity, electromagnetic shielding properties, and the influence of different fillers and filler amounts on rheological behavior. This work focuses on carbon black (CB), recycled carbon fibers (rCF), carbon nanotubes (CNTs), and complex nanomaterials. Therefore, silicon samples with different fillers and filler amounts were prepared using a dual asymmetric centrifuge and a three-roll mill. It has been found that even with small filler amounts, the electromagnetic shielding properties were drastically raised. The filler content as well as the dispersion technique have a significant influence on most of the fillers. It has also been found that the complex viscosity is strongly influenced by the dispersion technique as well as by the choice and amount of filler. In the experiments carried out, shielding values of over 20 dB were achieved with several fillers, whereby even 43 dB were reached with complex, pre-crosslinked fillers. This signal reduction of up to 99.99% enables almost complete shielding of the related frequency.

## 1. Introduction

Recent years have shown that, regarding electromagnetic interference, serious threats to electronic devices can occur. Whereas the influence on smart devices in the automotive sector is already critical, the interference with cardiac pacemakers or devices in a medical intensive care unit can be life-threatening [1,2,3,4,5]. To ensure the stability and functionality of any electrical device, EMI-shielding measures are applied. In many cases, metallic housings are used, which reflect incoming radiation. The disadvantage of metallic materials is the high reflectance of the irradiated radiation. The radiation reflected back in this way can, in turn, cause damage to the electrical components in surrounding, unshielded areas. Metallic housings and coatings, or the use of metallic components for EMI protection, are further defined by a high dead weight. The use of absorbing, elastic, lightweight materials is therefore to be preferred. To achieve these properties, the dispersion of carbon allotropes in adhesives or potting compounds is widely used.

The absorption mechanism in carbon-filled compounds is mainly justified by two mechanisms. Firstly, the convection current of free charge carriers inside a conductive material is induced by a changing external electromagnetic field. In the case of dispersed fillers inside a plastic matrix, the formation of a percolation network is preferable.

The second effect that affects the EM-shielding is the permittive losses. These occur when a dipole domain or (positive or negative) charged particles are exposed to an alternating electromagnetic field. According to the direction of the electromagnetic field, the domain’s alignment changes rapidly. Both effects are accompanied by a conversion of electrical energy into thermal energy.

Due to the heterogeneous material structure, the shielding characteristics of polymer-based nanocomposites deviate to some extent from those of homogeneous metals. With regard to the electrical properties of the nanocomposites, the conductive filler particles of the composites can be considered ohmic resistors or electrodes, and the polymer matrix can be considered dielectric. A corresponding composite material can thus be described as a network of interconnected resistors and capacitors in which the capacitors act as high-pass filters. This approach has been adopted by Saib et al. [6] For CB-modified polyethylene, the network model was simplified to a capacitor-resistor series circuit. Jeddi et al. [7] have performed similar studies for CB composites in a frequency range of up to 100 MHz. Below the electrical percolation threshold at 1 wt. % filler content, a frequency-dependent increase of the alternating current (AC) conductivity was measured, while above the threshold at higher filler contents, AC remained constant up to ca. 30 MHz.

**Figure 1:**
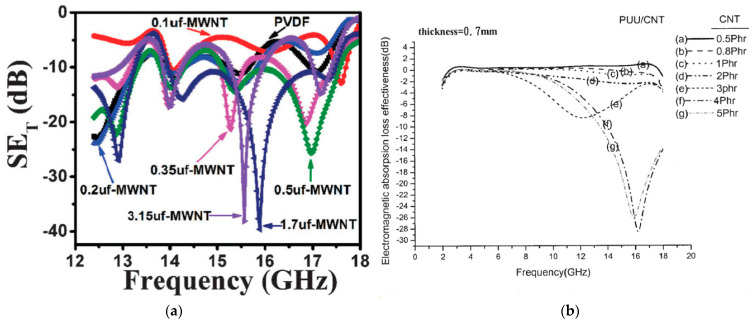
Representation of selective total shielding effectiveness (SE) when using CNT as filler, shielding with reversed sign definition of (**a**) MWCNT−filled Polyvinylidenflouride (PVDF) films of 0.3 mm thickness [12] and (**b**) CNT-filled polyurethane−urea (PUU) elastomer composites [13].

Higher filler contents are also associated with higher shielding capabilities [8,9]. With regard to different filler types, Al-Saleh et al. [10] have carried out a comparative study between CB, carbon nanostructures (CNS), and multi-walled carbon nanotubes (MWCNT). In correlation with the aspect ratio, the greatest shielding effect was found when using CNTs, and the weakest shielding was found with CB. Studies by Gupta et al. [11] have also shown that longer CNTs at constant diameter exhibit higher shielding. Furthermore, differences between single-walled carbon nanotubes (SWCNTs) and MWCNTs also show up. Due to the higher number of lattice defects, MWCNTs exhibit a higher permittivity, so they are said to have greater absorption and thus shielding [8]. Regarding frequency dependence, in addition to almost constant shielding characteristics (see Al-Saleh et al. [10]), strongly selective shielding behaviors are observed in the literature. Kumar et al. [12], as shown in Figure 1a, measured distinct peak expressions when unfunctionalized (uf)—MWCNTs were used, starting at a filler content of 0.5 wt. %. Similar observations have also been made by Ma et al. [13], where the peaks with increasing filler content in phr (parts per hundred parts of resin) vary not only in intensity but also in their frequency range (Figure 1b).

A further influence on the measured shielding is the selected specimen thickness. For example, Lecocq et al. [14] show that in the low-frequency range at 100 MHz, an increase in the layer thickness causes only a small improvement in the shielding, while in the high-frequency range at 8 GHz, the shielding increases proportionally to the sample thickness. Al-Saleh et al. [15] also investigated the thickness effect, and in the 8–12 GHz range, using 2.5 Vol-% CNT, an improvement in shielding from 6.7 dB to 10 dB with a thickness change from 1 mm to 2.8 mm occurred, which does not correspond to a proportional increase. This circumstance shows that a meaningful comparison of shielding effectiveness in the literature is difficult, especially in the case of different sample thicknesses, since influences such as the use of different dispersion methods also affect the shielding behavior [16]. Furthermore, most of the investigations concentrate on the X-band range (8–12 GHz), which is why reference values for lower frequency ranges are rarely found [17]. 

In contrast to other publications, this study gives an overview of different filler types and amounts in terms of rheological, electrical, and shielding properties by using a standard silicone and a unified mixing schedule together with homologated measurement conditions.

## 2. Materials and Methods

### 2.1. Base Material and Fillers

Sylgard 184 polydimethylsiloxane from Dow Inc., Midland, MI, USA, is used as the base material. According to the technical data sheet [18], this addition-curing two-component material are mixed in a ratio of 10:1 and cures at 25 °C within 48 h. Before this, however, fillers can be added to the system within the pot life of 90 min. The selected fillers are listed in Table 1 below.

Based on the state of the art, different filler products are investigated. In addition to the simplest allotrope of carbon, carbon black, recycled carbon fibers, and both single-walled carbon nanotubes and multi-walled carbon nanotubes are selected for the experiments.

### 2.2. Processing Methods for the Incorporation of Fillers

**Figure 2:**
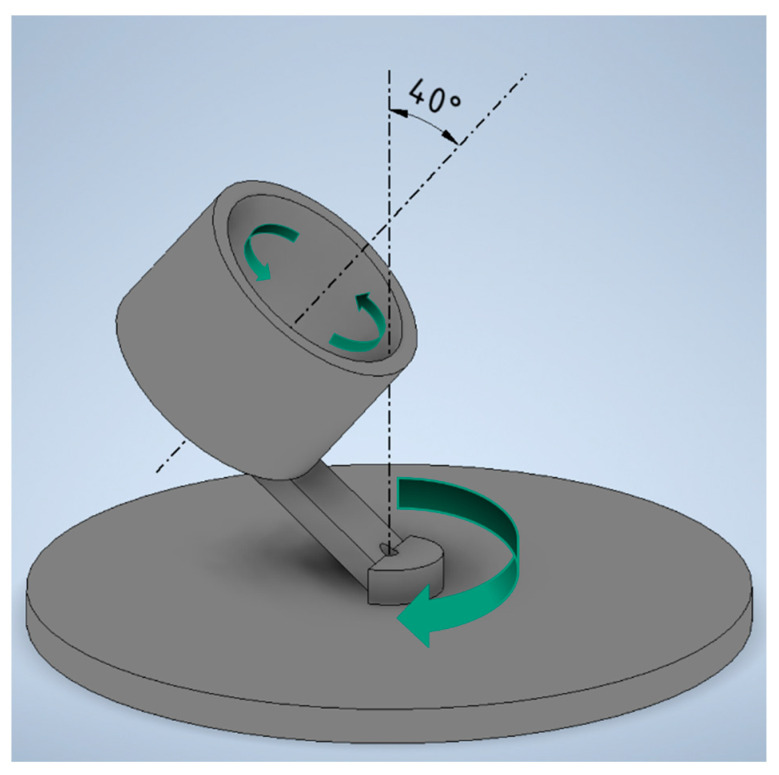
Functional principle of a dual asynchronous centrifuge.

For the manufacturing of the test specimens, the fillers are added to the base silicone resin in different proportions. Depending on the safety requirements, the components are first weighed in a glovebox or a fume hood using an AB204-S analytical balance from Mettler Toledo, Columbus, OH, USA, and then prepared in sealable polypropylene cups from Hauschild-Speedmixer, Hamm, Germany, for the following dispersion processes:

A dual asynchronous centrifuge (DAC) 150 from Hauschild-Speedmixer and a three-roll mill 80E from EXAKT, Norderstedt, Germany, are used for the dispersion process. In the DAC, a shear flow occurs, as shown in Figure 2, due to the superimposition of two counter-rotating centrifugal movements whose axes of rotation are tilted against each other as a result of the centrifugal forces acting. This shear flow can be used to disperse substances in the plastic cups utilized. 

After 60 s at 3500 revolutions per minute (rpm), a further dispersion step follows in the three-roll mill for the pre-dispersed substances. The speed ratio of 1:3:9 of the three rollers results in extremely high shear forces that lead to a homogeneous distribution of the fillers in the base matrix. In addition, the dispersion quality can be increased by multiple calendering runs, but Thostenson et al. [25] observed a degradation of the electrical properties in CNT/epoxy resin composites, which occurs with very intense calendering (up to 20 repetitions). This indicates an overstressing of the filler network formed or of the individual filler particles themselves, so that these are partially destroyed. The number of runs is therefore set at four and performed according to the list in Table 2.

### 2.3. Measurement Methods

#### 2.3.1. Rheological Measurement

The rotational rheometer C-VOR 150 from Malvern, Great Britain, is used for the rheological tests. It is equipped with a Peltier element, which maintains a constant temperature of 25 °C during the tests and is the lower support for the sample substrate. The upper support applies the normal force and torsional moment to the sample, which is placed in the measurement gap between them. For dispersions with low viscosity and high flow properties, the measuring gap is set at 1 mm. For samples with higher viscosity due to a higher filling content, a gap in the range of 1.4 mm to 1.5 mm is used. These are taken into account in the characteristic value calculation and therefore do not significantly influence the measurement results.

At the beginning of the experiment, an amplitude test is carried out with the unmodified silicone. This is conducted with a constant angular velocity of 10 rad/s for a range of 0.15 Pa to 60 Pa. A measured stress amplitude of 8 Pa in the linear viscoelastic range is determined, with which the frequency tests are then performed in a range of 0.1 rad/s to 100 rad/s.

For substrates with high complex viscosity, the shear stress selected at the beginning causes only very small shear amplitudes, which are at the lower sensitivity limit of the rheometer. To reduce the resulting measurement noise, a second amplitude of 60 Pa is used in these cases.

#### 2.3.2. Volume Resistance Measurement

To analyze and characterize the formation of electrical network paths in the resin of the base polymer caused by the fillers, the volume resistivity of the sample materials is measured. For this purpose, the sample materials must first be shaped to a suitable sample geometry. This is why the silicones filled with rCF and CB are drawn onto a PET carrier film using the Coatmaster 509 MC-III film applicator from Erichsen GmbH & Co. KG, Osterburken, Germany, to form a 200 µm thick film. The high-viscosity dispersions filled with NC7000, Tuball, and Miralon Pulp (MP) are produced with a thickness of 500 µm. The feed rate of the squeegee is 2.5 mm/s.

Afterwards, circular samples with a diameter of 80 mm are cut out of all the films produced in a laser cutting process.

The samples prepared can now be placed in the 8009 ring measuring cell from Keithley Instruments, Cleveland, OH, USA, combined with the 6517B electrometer. This setup corresponds to the resistance measurement of a real capacitor, in which the sample represents the dielectric. To prevent edge effects and the associated flow of charges over the sides of the dielectric, a setup with a protective electrode is used. 

According to the manufacturer, the measuring range of the measuring cell is limited to 10^3^–10^18^ Ω·cm. Test measurements have shown that volume resistivity below 10^8^ Ω·cm can no longer be reliably determined, which is why the range used is limited to 10^8^–10^18^ Ω·cm. For this setup, the test specimens must have a diameter of 62.5–101.6 mm, with a maximum specimen thickness of 3.2 mm.

A holding time of 30 s at a constant voltage of 40 V is selected for the experiment. Each measurement includes ten solid measurement points, whereby the first two measurement values are used for running-in and are not taken into calculation. Before the measurement is performed, the exact film thickness of the film sample, which is 80 mm in diameter, must first be determined. This is measured from the mean value of five different measuring points, which are taken at equal intervals around the circumference of the sample. After inserting the specimen, this value is stored in the associated testing software, after which the measurement is automatically carried out and evaluated.

For the determination of low contact resistances, a strip measuring cell is used in combination with the Multimeter 2000 from Keithley Instruments, Cleveland, OH, USA. In this setup, the strip sample is connected to the power source of the multimeter via the external source electrodes. The voltage drop across the sample is then measured by measuring the electrodes on the bottom side of the sample. The resulting resistance can be read on the multimeter. According to the principle of four-wire measurement, the influences of the cable and connection resistances are negated [26].

The strip samples used must have a minimum length of 45 mm and a maximum width of 20 mm, which is why a geometry of 50 mm × 18 mm is used, which can be cut from the test samples used in the ring measuring cell previously. To determine the contact resistance, the measured resistance R*_total_* is read off the multimeter.

To find the true resistance value R*_sample_*, the parallel-connected internal resistance of the measuring cell R*_in_* = 10^9^ Ω must be taken into account according to Equation (1). Then, in compliance with Equation (2), the specific volume resistance ρ*_DC_* can be calculated from the cross-sectional area A*_CS_* in combination with the electrode spacing d*_L_* of 15.6 mm.

(1) $$R_{sample}=\frac{1}{\left(\frac{1}{R_{total}}-\frac{1}{R_{in}}\right)}$$

(2) $$\rho_{DC}=R_{sample}*\frac{A_{cs}}{d_{L}}$$

#### 2.3.3. Measurement of Shielding Properties

Due to the observed differences in the viscosity of the dispersions, two different mold geometries are used for the fabrication of the EMI test specimens. Low-viscosity dispersions are poured into 8 mm × 2 mm × 25 mm cavities between two acrylic plates, which were previously screwed together. High-viscosity materials are poured into the cavities of an acrylic plate. In this case, the cover plate is pressed on and screwed in place after potting. Both geometries are designed slightly larger than required, allowing the final geometry to be cut after curing and any areas on the top of the mold with partial fillings or bubbles to be removed.

The test specimens are cured with the aid of a DEG-2 pressure compressor from Wassermann Dental, Hamburg, Germany. The test specimens are cured in this unit at 55 °C and 6 bar overpressure for 8 h. The continuous operating time of the device is limited to a maximum of one hour as standard. However, this limit was lifted on request by the manufacturer, bypassing the internal timer.

The characterization of the electromagnetic shielding behavior of the samples is performed with the aid of a stripline, which is connected to the vector network analyzer (VNA) FieldFox N9912A from Keysight, Santa Rosa, CA, USA, via two coaxial cables. The cables are Testline 5 LL test cables from the manufacturer Telegärtner Gerätebau GmbH, Klingenberg, Germany, which can be used up to 11 GHz and are terminated with N-connectors. Therefore, additional N-connectors to Sub-Miniature-Version-A-adapters are used for the connection to the stripline.

The test specimens are inserted with a positioning aid. For this purpose, the stripline is slightly spread, and the specimen is pushed under the septum and clamped. Relaxing the assembly results in a gapless connection between the sample and the line.

The FieldFox VNA has an impedance of 50 Ohm and a measuring range of 30 kHz to 6.5 GHz, which is used to the full extent for a frequency sweep. When evaluating the signals, the VNA divides the frequency range into individual frequency windows, which are used to calculate the S-parameters and thus determine the resolution. These are created by intermediate filters, which are available with bandwidths from 10 Hz to 100 kHz. For the experiments, a bandwidth of 1 kHz is used, which, with 11.2 s per sweep, offers a short measurement duration with high resolution. The output measuring points are set to 4001. Another parameter is the transmitting power used. This also has no relevant influence on the measurements and was left at the default setting of −15 dBm ≈ 0.03 mW. To reduce the electrical measurement noise of the line, a 3 dB attenuator is added to each of the coaxial cables.

For the characterization of the electrical shielding behavior, both the generation and the analysis of electromagnetic waves are necessary. In this context, a network can be described as a two-port network with an input and an output port. Through each port, an incoming (a) and an outgoing (b) electric voltage wave can pass. The scattering parameters S indicate the connection between the incoming and outgoing signals according to Equation (3). Since the waves have both an amplitude and a phase shift, the scattering parameters have a complex definition.

With the help of the VNA, an input wave can be directed into the two-port, and the reaction of the network can be described by the scattering parameters. The parameter S_11_ describes the reflection part so that the reflection shielding SE*_R_* can be determined according to Equation (4). The absorption shielding SE*_A_* is calculated by Equation (5) with the aid of the transmission parameter S_21_. The total shielding SE*_T_* is calculated as the sum of the absorption and reflection components according to Equation (6).

(3) $$\left(\begin{array}{c} b_{1}\\ b_{2} \end{array}\right)=\left(\begin{array}{cc} S_{11} & S_{12}\\ S_{21} & S_{22} \end{array}\right)\;\left(\begin{array}{c} a_{1}\\ a_{1} \end{array}\right)$$

(4) $${SE}_{R}=10\log\left(\frac{1}{1-{\left|S_{11}\right|}^{2}}\right)dB$$

(5) $${SE}_{A}=10\log\left(\frac{1-{\left|S_{11}\right|}^{2}}{{\left|S_{21}\right|}^{2}}\right)dB$$

(6) $${SE}_{T}={SE}_{R}+{SE}_{A}=10\;log\left(\frac{1}{{\left|S_{21}\right|}^{2}}\right)dB$$

## 3. Results

In the following subsection, the effect of fillers and filler content will be presented and discussed. First, the rheological result will give an overview of filler-induced influences on the flowability of the composite. Afterwards, the electrical properties will be analyzed regarding the volume resistivity. Lastly, the EMI-shielding properties are discussed and evaluated.

### 3.1. Rheological Results

**Figure 3:**
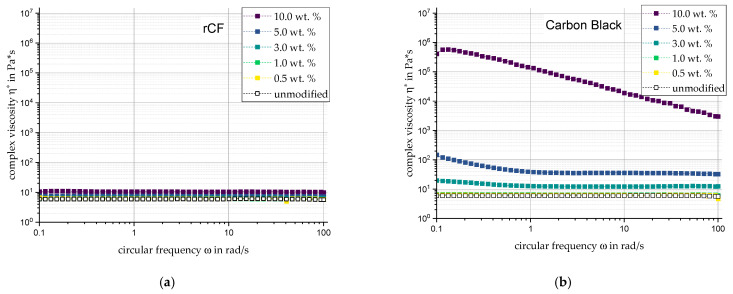
Complex viscosity of the low aspect ratio fillers (**a**) rCF and (**b**) CB in direct comparison and relation to filler content and circular frequency.

To make statements on the network structures formed and the flowability of the individual substrates, the complex viscosity is analyzed in the following ways: The influence of filler type and filler content will be considered. In this respect, it should be noted that the curing agent portion of the silicone, which accounts for 1/11 of the polymer weight content of the composite, is not taken into account. The least influence on the rheological properties is observed for the recycled carbon fiber in Figure 3a. The ideal viscous material behavior of the base polymer is maintained throughout, and the complex viscosity (at ω = 0.1 rad/s) is only doubled at 10 wt. % filler content with η* = 10.6 Pa·s. A significant increase in the dynamic modulus is also not observed. 

In contrast, Carbon Black in Figure 3b shows signs of network structures starting from a filler content of three percent. In the range from 0.1 rad/s to 1 rad/s, the flattening viscosity curves indicate shear thinning characteristics and incipient structural strength, which further develop with increasing filler content. At a filler content of 10 wt. %, an increase in the complex viscosity to 9.2 × 10^5^ Pa·s is observed. This indicates a rheological percolation range between 5 wt. % and 10 wt. % filler content.

**Figure 4:**
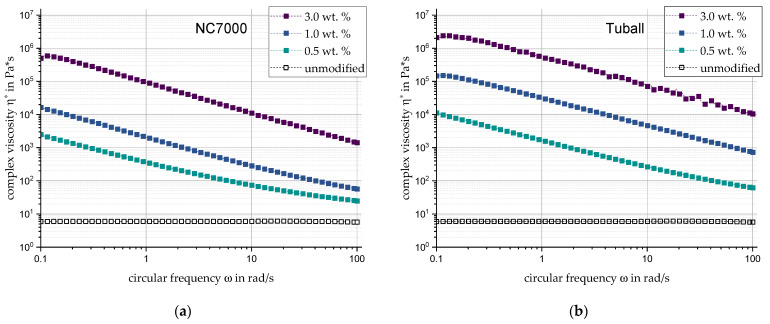
Complex viscosity of the CNTs (**a**) NC7000 and (**b**) Tuball in direct comparison and relation to filler content and circular frequency.

When NC7000 is used, already at the lowest filler content of 0.5 wt. %, the rheological percolation limit is exceeded (Figure 4a). The rheological properties for 3 wt. % are only minimally lower compared to 10 wt. % Carbon Black. For the filler Tuball, a developed network characteristic can be observed for each configuration in Figure 4b, so that a percolation limit below 0.5 wt. % filler content can be assumed. With η* = 2.11 × 10^6^ Pa·s the maximum complex viscosity at rest is already six orders of magnitude higher than that of the base polymer.

**Figure 5:**
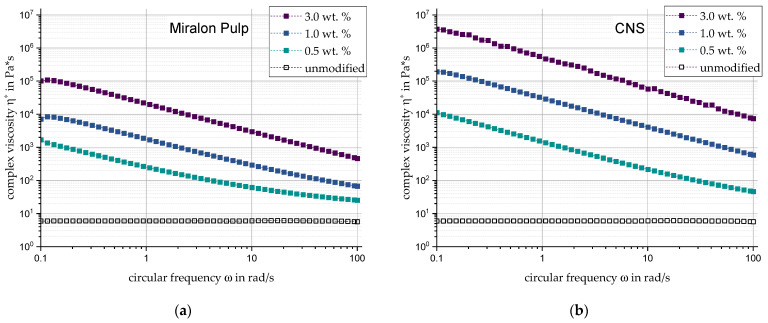
Complex viscosity of the complex carbon fillers (**a**) Miralon Pulp and (**b**) CNS in direct comparison and relation to filler content and circular frequency.

The rheological characteristics of the silicone filled with Miralon Pulp (Figure 5a) are basically similar to those of the Tuball composites. The differences can be found primarily in the generally lower values of the complex viscosity and the smaller viscosity changes between the filler content levels. The complex viscosity reaches a maximum of 1.01 × 10^5^ Pa·s. Athlos CNS shows, according to Figure 5b, the most significant property changes of all fillers considered. Once again, shear-thinning behavior can be observed.

### 3.2. Electrical Results

**Figure 6:**
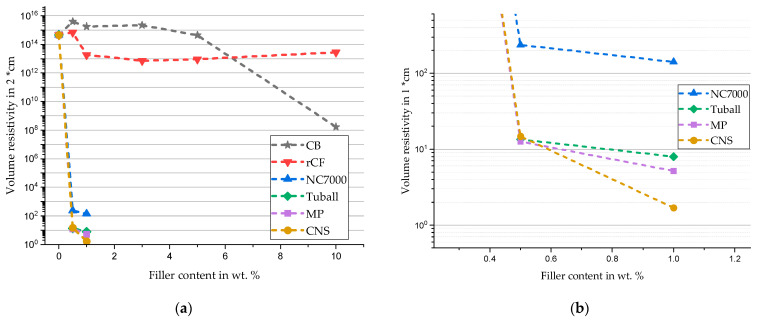
Volume resistivity for different carbon-based fillers and filler amounts (**a**) with a visible decrease of resistivity with rising filler content and (**b**) enlarged detailed view in a range of 0.5 wt. % and 1 wt. % filler content.

For the investigation of electrical percolation, the measured volume resistances are shown in the diagram in Figure 6a. In this respect, a clear subdivision of the measured resistances has emerged. When recycled carbon fiber is used, the volume resistance is consistently in a range between 10^13^ Ω·cm and 10^15^ Ω·cm, which corresponds to the unfilled silicone. For carbon black, on the other hand, a significant increase in electrical conductivity can be observed at a filler content of 10 wt. %. With a value of 1.7 × 10^8^ Ω·cm, the measured conductivity is close to the lower end of the sensitivity limit of the ring measuring cell. With regard to the CNT-based fillers, an electrically percolated material behavior is shown (Figure 6b). Composites filled with Miralon Pulp and Tuball show very similar volume resistivities, which are below those of NC7000. The lowest resistance is measured with the Athlos CNS at 1.69 Ω·cm. Compared to values from the literature, studies by Al-Saleh et al. [10] with a volume resistivity of 9 Ω·cm with 1 wt. % CNT-ABS show good agreement with the measured values.

### 3.3. EMI-Shielding Results

For the evaluation of the electromagnetic shielding, the shielding properties are calculated from the S-parameters of the specimens using Equations (4)–(6). For a simplified description, the values of each of the three same samples were combined into average curves.

**Figure 7:**
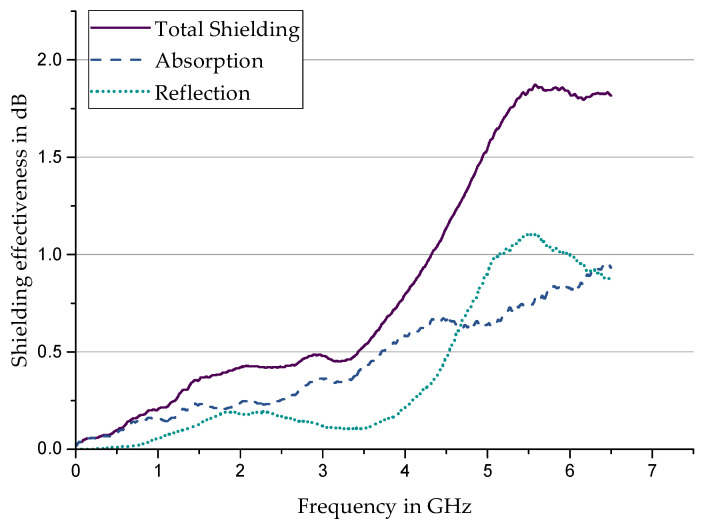
Shielding behavior of an unfilled Sylgard 184.

For reference, the shielding effectiveness of the unfilled silicone is shown in Figure 7. This shows a strong frequency dependence, which is, however, very low, with a maximum total shielding of 1.87 dB at 5.6 GHz. Both reflection and absorption generally increase with increasing frequency and are in the same order of magnitude.

In the following, the effects of the filler content and filler type on the frequency are investigated. For this purpose, the total shielding of the individual configurations of each filler is compared with each other.

**Figure 8:**
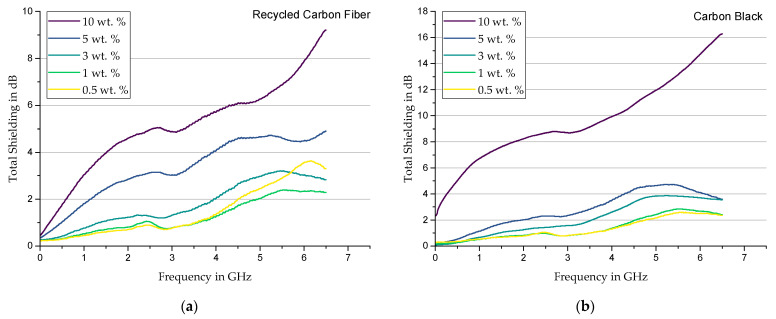
Total shielding of Sylgard 184 modified with (**a**) rCF and (**b**) Carbon Black.

**Recycled Carbon Fiber**—When using rCF; Figure 8a consistently shows a positive correlation between frequency or filler content and shielding. The curves indicate a similar filler characteristic of agglomerated rCF and carbon black. For example, both composites are characterized by a local minimum at values slightly above 3 GHz and a subsequent continuous increase in shielding properties.

**Carbon Black**—Figure 8b shows; in addition to the similar shielding behavior compared to rCF-filled samples, that a homogeneous distribution in specimens with a high CB content can result in fine network structures, which provide better shielding due to a large number of lossy conductive paths. This also finds good agreement with the rheological observations in Section 3.1. For this reason, the configuration with 10 wt. % filler content performs the best in shielding effectiveness.

**Figure 9:**
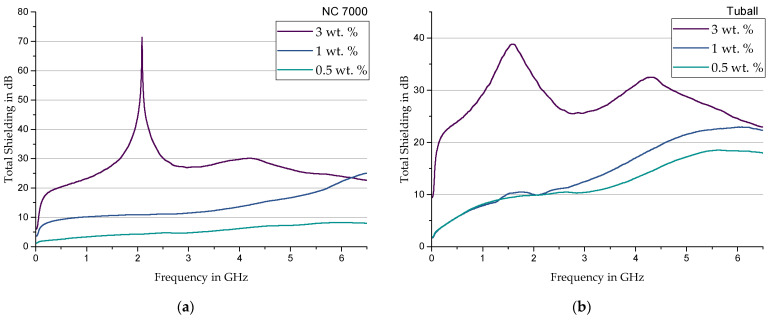
Total shielding of Sylgard 184 modified with (**a**) NC 7000 and (**b**) Tuball.

**NC7000**—For the use of MWCNT; a significant increase in shielding capability is seen compared to the previous fillers. Figure 9a shows a sudden increase in the range around 100 MHz from a filler content of 1%, whereupon the shielding increases steadily. Filler contents of 3 wt. % show very high selective shielding at 2.1 GHz with more than 70 dB. Furthermore, from a frequency of 4.2 GHz onward, a decreasing curve is observed. For measurements in a higher frequency range, Pawar and Biswas et al. [27] also found this decreasing trend for MWCNT composites. The shielding values of 20 to 25 dB determined by Pawar and Biswas et al. for frequencies above 8 GHz fit in seamlessly with the values determined in these measurements. Nevertheless, 3 wt. % filler content shows the best broadband shielding with over 20 dB in the range from 0.5 GHz to 6.5 GHz. 

**Tuball**—Compared to NC7000; the use of Tuball shows a significant increase in shielding for all configurations. The corresponding diagram is shown in Figure 9b. The highest filler content of 3 wt. % shows two global maxima at around 1.5 GHz and 4.2 GHz.

**Figure 10:**
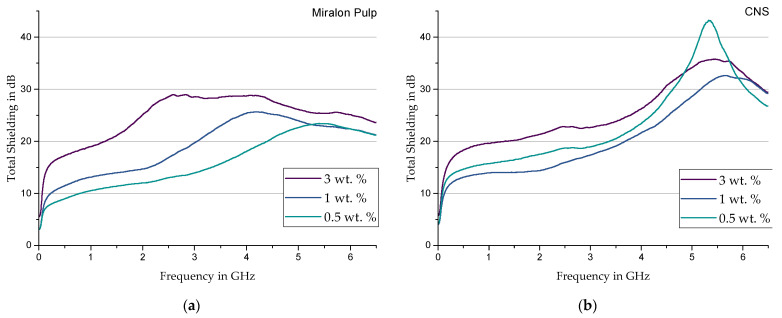
Total shielding of Sylgard 184 modified with (**a**) Miralon Pulp and (**b**) CNS.

**Miralon Pulp**—When using MP; the measurement curves (Figure 10a) for all configurations already show shielding values of more than 10 dB from a frequency of approx. 0.5 Hz. The selective shielding observed previously transitions to a range of 2 GHz to 3 GHz, depending on the filler content. Similar to the NC7000 filler, a slight drop in the shielding properties can also be observed here from a range between 4 GHz and 5 GHz. Due to the well-known correlation between electrical conductivity and shielding properties, the decreasing electrical resistances with increasing filler content found by Earp and Luhrs et al. [28] confirm the shielding values measured with Miralon Pulp.

**Athlos CNS**—The filler Athlos CNS shows in Figure 10b a relatively high shielding behavior already at 0.5 wt. % filler content. A peculiarity is the tendency of CNS samples with 0.5 wt. % filler content to show better shielding compared to the one-percent ones. One possible reason for this is the shear stress acting in the calendering process. Since this depends on the viscosity of the substrate used, at 0.5 wt. % filler content, a sufficiently favorable breakdown and thus expansion of the close-meshed network structure may have occurred, which has a positive effect on the shielding.

**Figure 11:**
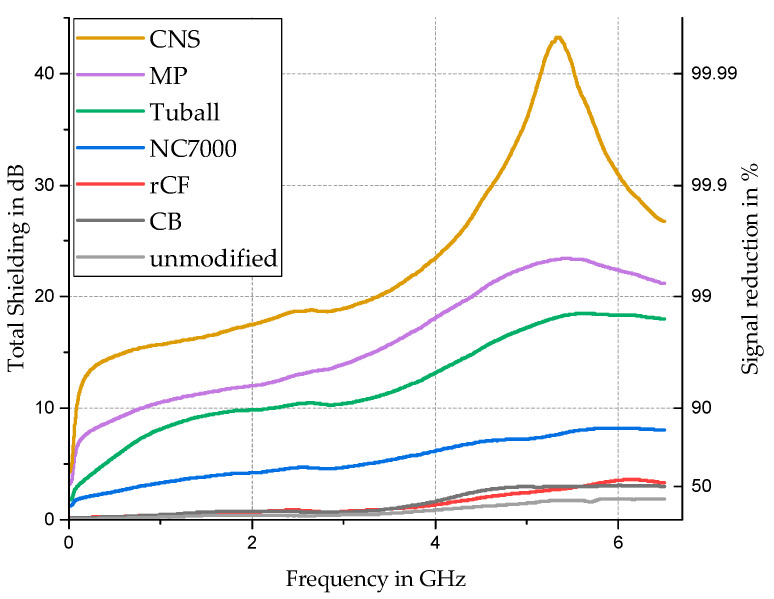
Comparison of frequency-dependent shielding in dB and signal reduction in per cent of filled silicone samples with a constant filler amount of 0.5 wt. % of different carbon-based fillers.

When comparing the shielding behavior of all composites with 0.5 wt. % in Figure 11, the simple carbon fillers (CB and rCF) show comparably low shielding, while the CNTs NC7000 (MWCNT) and Tuball (SWCNT) already have higher shielding values over the broad frequency spectrum. In addition, the MWCNTs have a lower shielding value than the SWCNTs. The complex carbon structures CNS and Miralon Pulp show overall the highest shielding values. Especially the CNS shows very high shielding between 15 dB and a peak value of 43 dB, at around 0.6 GHz to approx. 5.3 GHz. From this, the influence of the appearance of the carbon listed in Table 1 becomes particularly clear once again. In addition to the logarithmic unit system dB, the linear signal reduction in percent is also given for a better illustration of the results.

## 4. Discussion and Conclusions

The evaluation of the measurements has shown that the expression of the electromagnetic shielding behavior is accompanied by the formation of a filler network, which affects both the rheological and electrical aspects.

In summary, by comparing CB, NC7000, and Tuball, a lower percolation threshold at a higher aspect ratio was observed. Regarding the electromagnetic shielding capabilities, a significant frequency dependence was found for all the composites tested, according to the previous studies. In addition, a further explanation can be the approach of resonance phenomena within certain frequency ranges. Investigations into the increasing absorption due to resonances between the molecules within the matrix and the associated mathematical consideration as metamaterials were carried out by Li et al. in [29] and [30]. The periodically occurring maxima of the shielding in Figure 9, for example, are possibly due to resonances between the test specimens and the measuring cell. At these frequencies, antenna effects can occur, which allow energy to flow out of the measurement system. This loss cannot be analyzed by the measurement setup.

If the results presented here are considered in the context of the application as a shielding potting system, the demand for low viscosity and high volume resistivity (to avoid short circuits) results in a partly contradictory objective. Of the composites investigated here, the variant with calendered CB and 10% filler content still comes closest to representing a practicable compromise. With a shielding of 16.2 dB at 6.5 GHz, a significant improvement of about 15.5 dB is achieved compared to the unfilled silicone. However, the very high complex viscosity of 3 × 10^5^ Pa·s requires the use of diluting solvents or additional working steps such as puttying to ensure target-oriented processing.

The use of a low-electric conductive but shielding adhesive system, on the other hand, offers greater flexibility in terms of rheological and electrical properties. With regard to the lowest possible filler content, the composite with 0.5% CNS content offers the best shielding characteristics. Compared to all other variants with the same filler content, a very high shielding of 15.5 dB is already achieved at low frequencies of only 1 GHz. Furthermore, none of the other low-percentage variants achieves a shielding effectiveness of at least 25 dB, which is already present at frequencies of 4.2 GHz for the CNS-filled samples and reaches a maximum of 43 dB.

Due to the complex test matrix, selected fillers and filler contents have to be defined for future studies. Based on these, the electromagnetic shielding properties in higher frequency bands can subsequently be recorded. In addition, the findings obtained should also be tested and verified in series with other base polymer potting compounds. A combination of fillers is also considered promising. To transfer these advanced composites to practical applications, tests must be carried out on the long-term stability and durability of the key properties as well as the potential sedimentation phenomena of the materials. Furthermore, mechanical characterization tests on the composites allow a precise selection and adjustment of the materials for the respective application. Thanks to continuous manufacturing, no obstacles are expected during process scaling and improvement. Feedback from the first pilot applications should be used for further optimizations.

## Figures and Tables

**Table 1:** List of fillers used.

Filler	Manufacturer	Allotrope	Specific Surface Area in $m^{2}*g^{-1}$	Data Sheet
Recycled carbon fiber	R&G Waldenbuch, Germany	rCF	0.3 *^1^	[19]
Ensaco 350G	Imerys, Paris, France	CB	770	[20]
NC7000	Nanocyl, Sambreville, Belgium	MWCNT	250–300	[21]
Tuball	OCSiAl, Luxembourg	SWCNT	500–1000	[22]
Miralon Pulp	Huntsman, Salt Lake City, UT, USA	n.a.	200	[23]
Athlos CNS	Cabot, Boston, MA, USA	n.a.	200	[24]

*^1^ Calculated from the material properties in the datasheet.

**Table 2:** Parameters of the calendering process.

Run	Shear Gap 1 in µm	Shear Gap 2 in µm	Number of Revolutions *n*_3_ in min^−1^
1	90	30	90
2	60	20	90
3	30	10	90
4	15	5	90

## Data Availability

The data presented in this study are available on request from the corresponding author.
